# Real-Time Adaptive Motion Management Using CyberKnife Synchrony™ for Lung Stereotactic Body Radiotherapy (SBRT): A Case Report

**DOI:** 10.7759/cureus.83803

**Published:** 2025-05-09

**Authors:** Jiaqi Gao, Sheng Zhang, Jun Han, Jing Yang

**Affiliations:** 1 Cancer Center, Union Hospital, Tongji Medical College, Huazhong University of Science and Technology, Wuhan, CHN; 2 Proton Therapy, Union Hospital, Tongji Medical College, Huazhong University of Science and Technology, Wuhan, CHN

**Keywords:** cyberknife, fiducial-free tracking, non-small cell lung cancer, nsclc, real-time adaptive radiotherapy, sbrt, stereotactic body radiotherapy, synchrony

## Abstract

Real-time adaptive motion management is critical for delivering precise stereotactic body radiotherapy (SBRT) to lung tumors while sparing adjacent organs-at-risk. This case report describes the successful application of the CyberKnife Synchrony™ Respiratory Tracking System (Accuray Inc., Sunnyvale, CA, USA) in a patient with early-stage non-small cell lung cancer (NSCLC). A 70-year-old male with an NSCLC lesion in the left upper lobe underwent SBRT with real-time fiducial-free tracking. The total prescribed dose was 48 Gy in four fractions. Post-treatment imaging confirmed local control with no acute toxicity. This report highlights the clinical feasibility of CyberKnife®-based real-time adaptive motion management for lung SBRT. These practical insights are valuable for centers adopting this technology.

## Introduction

Lung cancer is one of the most common and aggressive malignancies worldwide, with a high mortality rate [1]. Radiotherapy plays a crucial role in the treatment of lung cancer, especially for patients who are not suitable for surgery. However, the movement of lung tumors during respiration poses a significant challenge to the precision of radiotherapy. Real-time adaptive motion management is a promising approach to address this issue, improving the accuracy and effectiveness of radiation delivery. Lung tumor motion due to respiration poses significant challenges in radiotherapy, necessitating motion management to minimize planning target volume (PTV) margins and reduce organ-at-risk (OAR) doses. The CyberKnife® VSI™ Robotic Radiosurgery System with Synchrony™ Respiratory Tracking System (Accuray Inc., Sunnyvale, CA, USA) enables real-time fiducial-free tumor tracking by correlating external surrogates with internal tumor motion. This case report presents a patient with inoperable lung cancer who underwent stereotactic body radiotherapy (SBRT) using the CyberKnife® System with real-time adaptive motion management.

## Case presentation

Patient background

**Figure 1:**
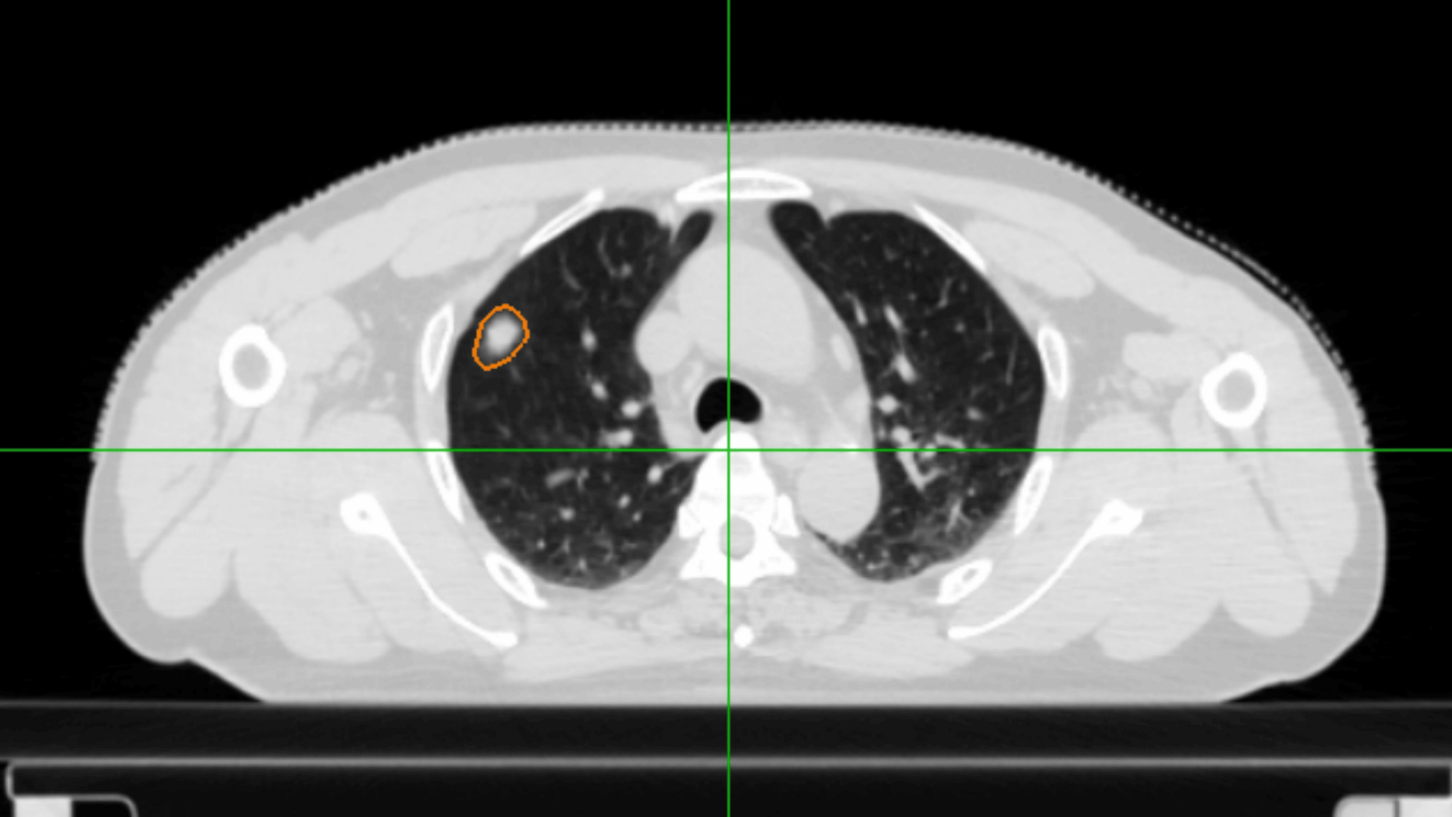
Axial CT showing a 15-mm NSCLC lesion in the left upper lobe CT: computed tomography, NSCLC: non-small cell lung cancer

A 70-year-old male patient presented with a 15-mm nodule in the left upper lobe (Figure 1) detected by a CT scan in January 2025, with no evidence of other abnormal uptake. A biopsy confirmed the diagnosis of non-small cell lung cancer (NSCLC) stage I. Due to poor pulmonary reserve, SBRT was recommended over surgery.

Treatment plan

The PTV was created by adding 5 mm margins to the gross tumor volume in all directions. Synchrony™ (fiducial-free tracking with an external light-emitting diode (LED) marker correlated to internal tumor motion), integrated with the CyberKnife® System, was used to track the tumor in real-time during treatment (continuous correlation model updates). This system allows for continuous adjustment of the radiation beam to compensate for respiratory motion, ensuring precise radiation delivery to the tumor while minimizing exposure to surrounding healthy tissues. The prescription dose was 48 Gy in four fractions (PTV coverage: D95 = 98%). The critical organ dose limits used in radiotherapy planning are based on the study by Timmerman [2]. The OAR constraints included a maximum heart dose (Dmax) of less than 30 Gy and a lung V20 of less than 10%. Gamma analysis showed a passing rate greater than 95%, with results meeting the 3%/2 mm criteria at 98.2%. The treatment time for each fraction was 47 ± 3 minutes.

**Figure 2:**
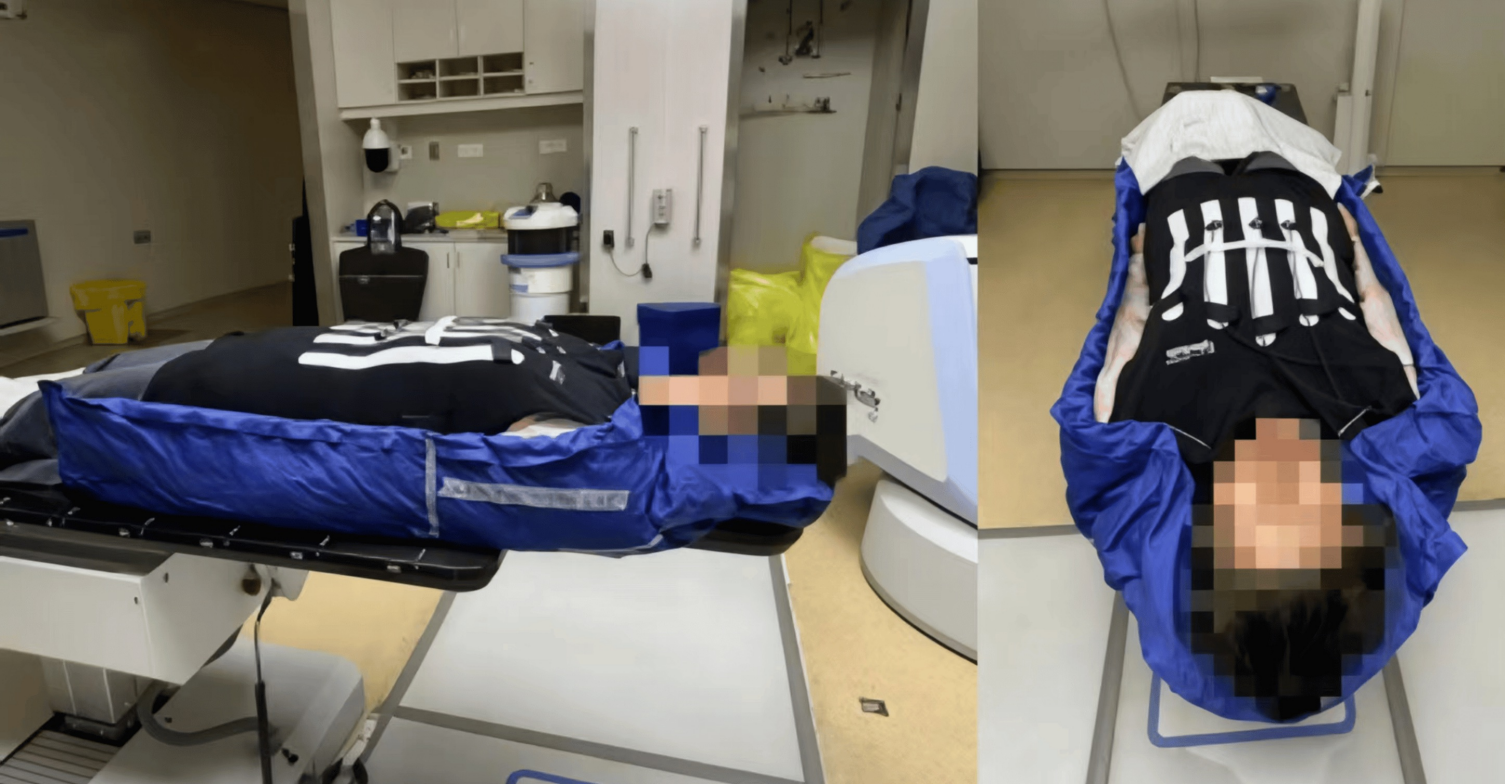
Real-time tracking with abdominal LED marker LED: light-emitting diode

Pre-treatment motion simulation revealed irregular breathing patterns, and audio coaching improved respiratory regularity. Synchrony™ tracked the tumor using an external LED marker on the abdominal wall (Figure 2). Treatment time averaged 47 minutes per fraction.

Treatment results

**Figure 3:**
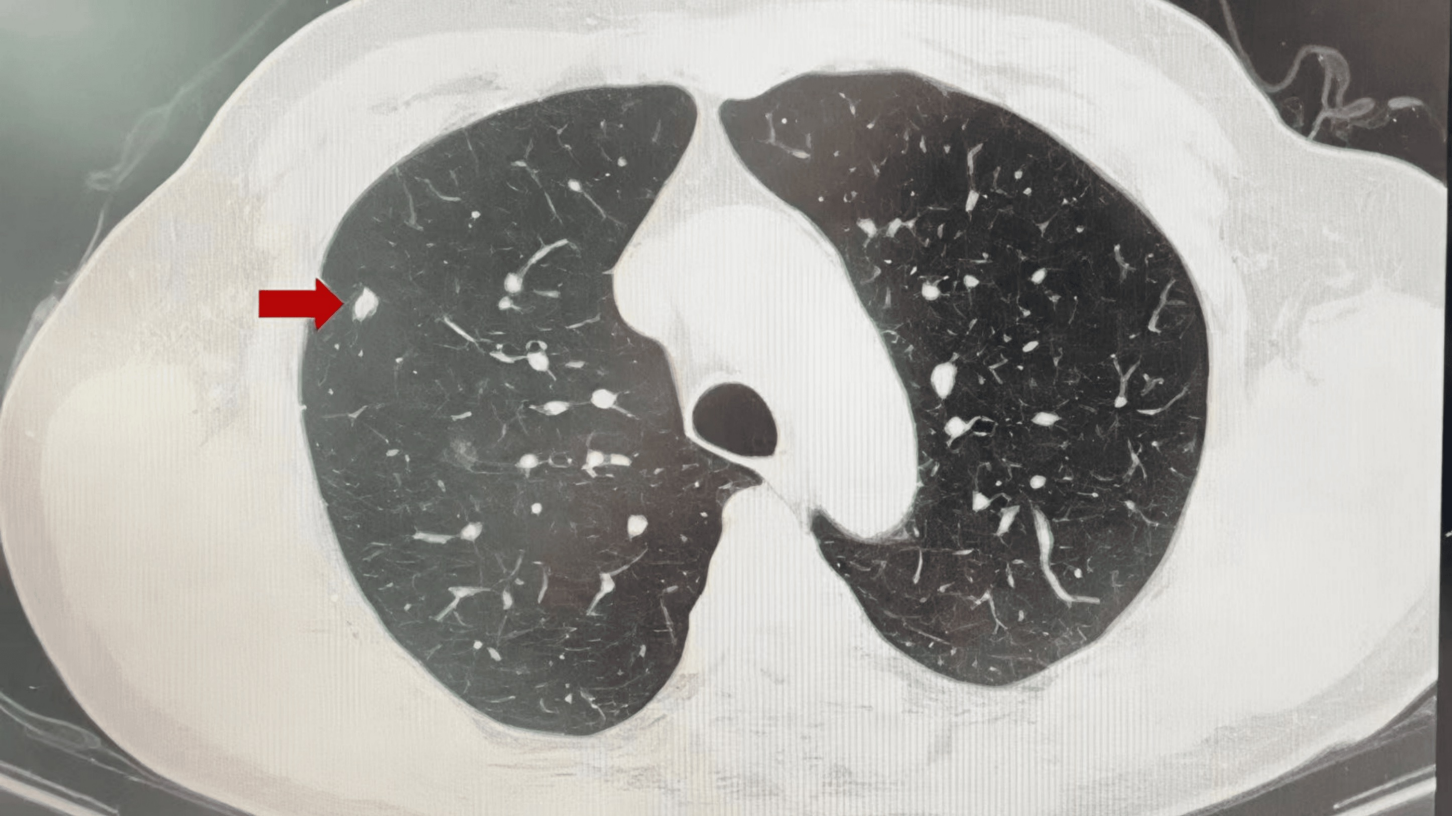
Follow-up imaging at three months

All four fractions were completed without interruptions. The gamma passing rates of 2D doses were within the clinically acceptable range. The detection accuracy of the tracking target was within a 1.0 mm margin in the phantom validation. The treatment was completed without major complications, and the patient tolerated the procedure well. No acute toxicities (grade 0 pneumonitis and esophagitis) were observed, and pulmonary function was maintained. Follow-up imaging at three months showed stable disease and reduced tumor size (5 mm) on CT (Figure 3). No radiation fibrosis on CT was found.

## Discussion

This case demonstrates adaptability to challenging scenarios (e.g., irregular breathing) and fiducial-free precision, which remains underreported in smaller centers. Key findings highlight the benefits of real-time tracking, including reduced PTV margins compared to cases without tracking. Specifically, a 5 mm margin was achieved, minimizing lung V20 to 8%. Submillimeter tracking accuracy ensured high precision, with target coverage reaching D95: 98% and improved sparing of OARs relative to non-tracked cases. Although treatment times were prolonged, workflow efficiency was preserved, and therapists maintained patient compliance through audio coaching.

Comparisons with non-tracking SBRT (historical cohort) revealed superior dosimetry and reduced toxicity [3-5]. Synchrony™ allowed for precise delivery of high-dose radiation to the tumor while minimizing exposure to surrounding healthy tissues. PTV margins were minimized, reducing dose to critical structures like the heart and healthy lung tissue [6,7]. This approach can potentially improve local control rates and reduce treatment-related toxicities. Future studies should further investigate this technique's long-term outcomes and benefits in a larger patient population. CyberKnife Synchrony™ facilitates safe and precise lung SBRT through real-time adaptive motion management.

## Conclusions

This case report provides evidence of the clinical utility of real-time adaptive motion management in lung cancer treatment using the CyberKnife® System. Integrating Synchrony™ with the CyberKnife® technology ensures clinical safety and efficacy, making it a valuable tool in managing lung cancer patients.
